# The Impact of Increased Maternal sFlt-1/PlGF Ratio on Motor Outcome of Preterm Infants

**DOI:** 10.3389/fendo.2022.913514

**Published:** 2022-06-30

**Authors:** Lisa Middendorf, Alexandra Gellhaus, Antonella Iannaccone, Angela Köninger, Anne-Kathrin Dathe, Ivo Bendix, Beatrix Reisch, Ursula Felderhoff-Mueser, Britta Huening

**Affiliations:** ^1^Department of Pediatrics I, Neonatology, Pediatric Intensive Care and Pediatric Neurology, University Hospital Essen, University of Duisburg-Essen, Essen, Germany; ^2^Department of Obstetrics and Gynecology, University Hospital Essen, Essen, Germany; ^3^Department of Obstetrics and Gynecology, University of Regensburg, Clinic St Hedwig of the Order of St. John, Regensburg, Germany; ^4^Centre for Translational Neuro- and Behavioural Sciences, C-TNBS, Faculty of Medicine, University Duisburg-Essen, Essen, Germany; ^5^Department of Health and Nursing, Occupational Therapy, Ernst-Abbe-University of Applied Sciences, Jena, Germany; ^6^Department of Pediatrics I, Neonatology and Experimental Perinatal Neurosciences, University Hospital Essen, University Duisburg-Essen, Essen, Germany

**Keywords:** motor outcome, prematurity, infant, sFlt-1, PIGF, MOS-R

## Abstract

**Background:**

The sFlt-1 (soluble fms-like tyrosine kinase-1)/PlGF (placental growth factor) ratio serves as a clinical biomarker to predict the hypertensive, placenta-derived pregnancy disorder pre-eclampsia which is often associated with placental dysfunction and fetal growth restriction. Additionally elevated levels also indicate an increased risk for prematurity. However, its predictive value for subsequent neonatal neurological outcome has not been studied.

**Objective:**

This study aimed to evaluate the correlation of maternal sFlt-1/PlGF ratio with early motor outcome of preterm infants. Design/Methods: 88 preterm infants (gestational age ≤ 34 + 0) born between February 2017 and August 2020 at the Department of Obstetrics and Gynecology, University Hospital Essen in Germany, were included, when the following variables were available: maternal sFlt-1/PlGF levels at parturition and general movement assessment of the infant at the corrected age of 3 to 5 months. The infants were stratified into high and low ratio groups according to maternal sFlt-1/PlGF cut-off values of 85. To investigate the early motor repertoire and quality of spontaneous movements of the infant, the Motor Optimality Score (MOS-R) based on antigravity movements and posture patterns, was applied. In the given age, special attention was paid to the presence of fidgety movements. Linear regressions were run to test differences in infants motor repertoire according to the maternal sFlt-1/PIGF ratio.

**Results:**

Linear regression analysis showed that the sFlt-1/PlGF ratio does not predict the MOS-R score (β=≤0.001; *p*=0.282). However, children with birth weight below the 10th percentile scored significantly lower (mean 20.7 vs 22.7; *p*=0.035). These children were 91% in the group with an increased ratio, which in turn is a known predictor of low birth weight (β= -0.315; *p <*0.001). In the group with a high sFlt-1/PLGF ratio above 85 the mothers of female infants had a lower average sFlt-1/PlGF ratio compared to a male infant (median: 438 in female vs. 603 in male infant, *p*=0.145).

**Conclusions:**

In our cohort, especially low birth weight, which correlated with an elevated sFlt-1/PlGF ratio, had a negative effect on the outcome in the MOS-R. A direct correlation between an increased ratio and a worse motor outcome was not demonstrated.

## Introduction

Preeclampsia (PE) is a hypertensive pregnancy disorder that occurs in approximately 2% to 4% of all pregnancies and is accompanied by substantial morbidity and mortality for both mother and infant and long-term risks for chronic diseases (1). The exact causes of PE are still unclear, but contributors are systemic endothelial dysfunction, impaired angiogenesis, and decreased vascular compliance (2, 3). In recent years, research has focused primarily on soluble anti-angiogenic factors, such as soluble fms-like tyrosine kinase-1 (sFlt-1) and pro-angiogenic factors, such as placental growth factor (PlGF). An anti-angiogenic imbalance upon increased sFlt-1 levels has been shown to be a critical factor in the pathogenesis of placentally associated disorders such as PE and is strongly associated with the crucial process of remodelling of the maternal spiral arteries (4, 5). As an anti-angiogenic factor on the surface of vascular endothelial cells, sFlt-1 blocks the responses of the pro-angiogenic factors vascular endothelial growth factor (VEGF) and PlGF (6). Those changes in the concentration of angiogenic factors can be measured in maternal blood samples – an elevated sFlt-1/PlGF ratio can occur weeks before clinical symptoms of PE arise (7).

Therefore, measurement of the sFlt-1/PlGF ratio has become an established marker in clinical practice for early prediction of PE. Previous studies showed that patients with a ratio >85 are at high risk for developing PE as well as placental insufficiency (8). In combination with blood pressure measurements and uterine artery doppler a PE diagnosis can often be made at an early stage, resulting in lower maternal morbidity (9, 10). The only effective therapy to cure PE so far is the removal of the placenta and consequently delivery of the baby at any gestational age. Approximately 15 percent of all preterm births are due to PE (11). Previous studies demonstrated that an increased sFlt-1/PlGF ratio was directly associated with an increased risk of preterm birth, reduced APGAR scores and fetal growth retardation (FGR) (12, 13). Recently it could be shown that in cases of FGR the fetal well-being, as measured by feto-maternal Doppler parameters and the severity of the placental dysfunction, as measured by the urgency of delivery, is reflected by the level as well as the increase in the sFlt-1/PlGF ratio in the maternal serum during pregnancy (14). Preterm birth, especially in combination with FGR, resulted in a greater risk for children to develop neurological and cognitive deficits in their later life (15, 16). A longitudinal study of 284 very preterm infants (<33 weeks) showed that the infants with FGR had significantly lower cognitive and motor scores in the Bayley Scales of Infant and Toddler Development at corrected age (CA) of 22 month compared with appropriate for gestational age (GA) infants (16). There is no evidence to date on early motor outcome particularly in relation to maternal sFlt-1/PIGF ratio.

To evaluate early motor development this study uses the Motor Optimality Score – Revised (MOS-R) of General Movements at the CA of 3-5 months (17). The score is based on the Prechtl General Movement Assessment, which is orientated on visual Gestalt perception of video-recorded age-specific normal or abnormal general movement patterns. Age-appropriate movements are the so-called fidgety movements (FM). These continuous and tiny movements and rotations are predominantly found in the shoulders, wrists, hips and ankles, are of small-amplitude and of moderate speed. Abnormal movements, especially the absence of FM, are highly associated with the development of severe neurological deficits (18). In addition to the classification of general movements, the MOS-R evaluates posture and movement patterns, as well as movement repertoire and character (19, 20). The score has a high sensitivity for the diagnosis of cerebral palsy and can also provide information about its prognosis, type and severity (17). A recent study of 53 extremely preterm infants by Örtqvist et al. evaluate the early motor outcome and the overall neurodevelopment at 12 years of age (21). The GMA and MOS-R predict the later adverse neurodevelopment with high positive predictive value, specificity, and sensitivity.

Previous studies have focused primarily on the short-term outcome of elevated sFlt-1/PlGF levels on preterm infants such as birth weight, APGAR, and neonatal morbidity, but its predictive value for subsequent neurological outcome has not been examined so far (12, 13). In this study, we aim to evaluate the correlations between maternal sFlt-1/PlGF ratio with early motor outcome of preterm infants at the age of 3 to 5 months.

## Material and Methods

### Study Population

**Figure 1:**
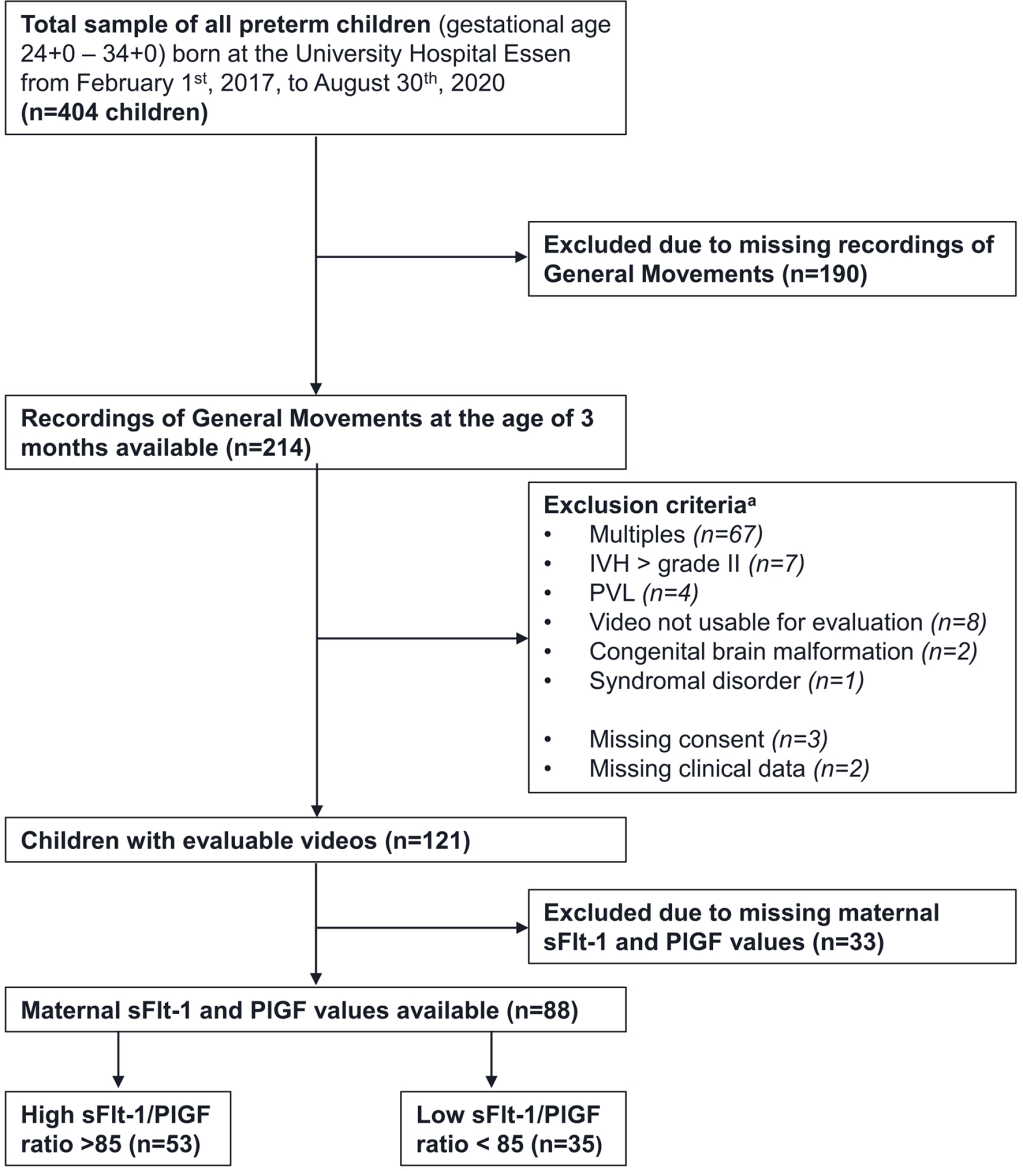
Recruitment strategy and exclusion criteria. Notes. IVH= intraventricular hemorrhage, PVL= periventricular leukomalacia, ^a^ participants can fulfill multiple exclusion criteria.

88 preterm infants (GA 24 – 34 weeks) born between February 2017 and August 2020 at the Department of Obstetrics and Gynecology, University Hospital Essen, Germany, were included. By using a maternal sFlt-1/PlGF cut-off ratio of 85 the infants were separated into two groups (8). The patients were selected according to the following criteria (1): the maternal sFlt-1 and PlGF levels were available (2); general movement assessment (GMA) of the infant at the CA of 11-16 weeks was performed of sufficient quality (3); newborns of single-fetus pregnancies (4); GA at birth between 24 to 34 + 6 weeks. Exclusion criteria are: periventricular leukomalacia, intraventricular hemorrhage (IVH) > grade II, genetic syndromes, congenital malformations, and verified congenital infections. 190 of the children born in the mentioned period had to be excluded due to missing video recordings. **Figure 1** gives an overview about inclusion and exclusion criteria. Gender differences were assessed using appropriate statistical test designs. PE and FGR were diagnoses according to current guidelines (22).

### Ethics

The study is approved by the local ethical committee in Essen (18-8388-BO, 12-5212-BO). Pregnant women and parents of all participants gave written informed consent.

### Sampling of Blood Serum and Determination of sFlt-1 and PlGF

Blood samples (9 ml) were collected between week 20 and 34 of gestation using S-Monovettes (Sarstedt AG & Co., Nümbrecht, Germany), stored at 4° C, and processed within 4 hours to avoid blood cell lysis. Blood fractionation was carried out by centrifugation for 10 min at 2500 rpm. Three to four milliliters of the upper phase, constituting blood serum, was removed, stored at -80° C and subjected to the determination of sFlt-1, and PlGF.

50 μl of the pre-diluted sample plus 150 μl of dead volume was used to measure concentration of sFlt-1 (BRAHMS sFlt-1 KRYPTOR), PlGF-Plus (BRAHMS PlGF plus KRYPTOR), all from Thermo Fisher Scientific, using BRAHMS KRYPTOR compact PLUS machine based on TRACE^®^ Technology (Time-Resolved Amplified Cryptate Emission) (Thermo Fischer Scientific, BRAHMS GmbH, Hennigsdorf, Germany), according to the protocol.

The detection limit was assessed as being 22 pg/mL for sFlt-1 and 3.6 pg/mL for PlGF. The functional assay sensitivity, detected by inter-assay precision of 20% coefficient of variability (CV), has been assessed as being lower than 29 pg/mL for sFlt-1 and 6.7 pg/mL for PlGF.

### Clinical Characteristics

Prenatal, perinatal, and postnatal clinical characteristics were collected retrospectively from the medical records of infants and mothers and from the clinic’s own databases. On the maternal side, information on pregnancy-related diagnostic procedures and therapies were collected in addition to clinical data such as parity and maternal and gestational age. The following causes of preterm delivery were noted: infections, premature rupture of membranes (PROM), gestational diabetes, PE, Eclampsia, FGR and HELLP-Syndrome (Hemolysis, Elevated Liver enzymes and Low Platelets). For the children the presence of diseases such as necrotizing enterocolitis, bronchopulmonary dysplasia (BPD), sepsis, retinopathy of prematurity (ROP) and IVH was recorded. Furthermore, information on body measurements, GA, birth details, diagnosis - and therapy methods were obtained.

Between 24 and 33 weeks of gestation, ultrasound diagnostics were performed, which included the determination of the following parameters: Pulsatility Index (PI) of the uterine arteries (UtA), PI of the umbilical artery (UA), PI of the middle cerebral artery (MCA), the cerebroplacental ratio (CPR) which is the ratio between the PI of the MCA and the UA (23, 24) and PI of the Ductus venosus (DV) (data of DV not shown).

### Motor Optimality Score

To assess MOS-R, we used pre-existing videos of the children, which were obtained during the routine GM evaluation at 3-5 months of CA. Prior to this, the parents were informed about the assessment and their written consent was obtained. The video recordings followed the guidelines of Prechtl’s GM Assessment. Infants need to be awake and active for at least 3 minutes; periods of crying, fussiness, and sucking on a pacifier were excluded (18, 25). The evaluation was performed by two GM Trust certified advanced scorers blinded to the infants’ medical histories and neurodevelopmental outcomes. According to the evaluation sheet, the FM are assessed first. These can be scored as continual, intermittent, sporadic/absent, or abnormal exaggerated (18, 19). In the second step the movement and posture patterns (23 and 13 items) are assessed in detail as well as movement character (9 items) (26). Finally, points are awarded for following five subcategories: (i) FM, (ii) age-adequacy of motor repertoire, (iii) quality of movement patterns other than FM, (iv) posture, and (v) movement character. 5 to 28 points can be achieved, with 28 points being the best possible value (17, 18). MOS-R from 25 to 28 is classified as optimal, 20–24 is mildly reduced, 9–19 is moderately reduced and 5–8 is severely reduced (26–28).

### Statistical Analysis

Statistical analysis was performed by using SPSS statistics, IBM Ehningen, version 26. The Kolmogorov-Smirnov test was used to test the normality of distribution of values. For comparing two independent groups the nonparametric Mann-Whitney-U test was used for abnormal distribution and the parametric t test was used when the values presented normal distribution. The chi square or Fisher exact tests was utilized to compare cross-tabulated data and a two-tailed significance level of 0.05 was established. To determine a correlation (e.g., between birth weight and sFlt-1/PlGF Ratio) the Pearson or Spearman correlation coefficient was used depending on whether a normal distribution was present. Linear regressions were run to evaluate relationships between motor outcome and sFlt-1/PlGF ratio.

## Results

Maternal conditions such as HELLP, FGR and PE were significantly more common as expected in the group with an increased sFlt-1/PlGF ratio. In addition, these mothers had significantly higher blood pressure values prenatally. Prenatal diagnosis showed a reduced CPR and an increased PI of the umbilical artery in the high-ratio group (s. **Table 1**). The sFlt-1/PlGF ratio correlated significantly positively with the PI of both uterine arteries (*p_right_=*0.031; *p_left_* <0.001), the PI of the umbilical artery (*p*<0.001) and negatively with the PI of the middle cerebral artery (*p*=0.010) and the CPR (*p*=0.002) (s. **Table 2**). The Pearson Correlation test showed a positive trend between CPR and MOS-R-Scores. (r=0.206; *p*=0.099).

**Table 1:** Maternal data.

	High sFlt-1/PlGF ratio (n=53)	Low sFlt-1/PlGF ratio (n=35)	*p^a^*
Age	31 [18-42]	32 [18-45]	
Blood Pressure			
Systolic	144 [33]	125 [21]	**<0.001**
Diastolic	85 [21]	71 [7]	**<0.001**
Morbidity			
PE	33 (62.35)	1 (2.9%)	**<0.001**
Eclampsia	1 (1.9%)	0	0.414
HELLP	9 (17%)	1 (2.9%)	**0.004**
FGR	33 (62.3%)	5 [(14,3%)	**<0.001**
Gestational diabetes	5 (9.4%)	5 (14.3%)	0.483
PROM	7 (13.2%)	8 (22.9%)	0.229
Infection/Fever	2 (3.8%)	8 (22.9%)	**0.006**
Ultrasound diagnostics			
PI left uterine artery	1.51 [1.23]	1.23 [0.75]	0.254
PI right uterine artery	1.34 [1.24]	1.29 [0.73]	0.626
PI umbilical artery	1.48 [0.38]	1.13 [0.59]	**0.009**
PI middle cerebral artery	1.72 [0.46]	1.82 [0.86]	0.389
Cerebroplacental ratio	1.36 [0.77]	1.73 [1.32]	**0.012**
Medication			
Tocolysis	10 (18.9%)	13 (37.1%)	0.056
Fetal lung maturation	47 (88.7%)	33 (94.3%)	0.916
Apheresis*	3 (5.8%)	0	0.152

Data are given as n (%) or mean [IQR]. PE, preeclampsia, HELLP, HELLP-syndrome (haemolysis, elevated liver enzyme levels, low platelet count), FGR, fetal growth restriction, PROM, premature rupture of membrane, PI, pulsatility Index. ^a^p values are calculated using Pearson’s chi-square test and Mann-Whitney U-test. *Therapeutical plasma exchange, sFlt-1/PlGF ratio did not fall below 85.

Bold results indicate a p-value less than the significance level of 0.05.

**Table 2:** Correlation analysis between various ultrasonography parameters and the maternal sFlt-1/PlGF ratio upon study cohort.

Parameter	sFlt-1/PlGF ratio upon study cohort (n=88)	
	Correlation coefficient	*p^a^*
PI left UtA	0.527	**<0.001**
PI right UtA	0.309	**0.031**
PI UA	0.415	**<0.001**
PI MCA	-0.299	**0.010**
CPR	-0.373	**0.002**

Flt-1, soluble Fms-like tyrosine kinase 1, PlGF, placental growth factor, PI, pulsatility index, UtA, uterine artery, UA, umbilical artery, MCA, middle cerebral artery, CPR, cerebroplacental ratio. ^a^p values are calculated using the Spearman’s rank correlation test.

Bold results indicate a p-value less than the significance level of 0.05.

The children groups did not differ significantly regarding gender distribution, GA at birth and GA at sFlt-1/PlGF determination, and age at follow-up (s. **Table 3**). The children in the high ratio group had a significantly lower birth weight (mean 1120 g vs. 1550 g, *p*=0.004). Even at the 3-month follow-up, the group with elevated maternal sFlt-1/PlGF ratio showed significant differences in body weight (mean 5495 g vs. 6192 g, *p*<0.001), body length (mean 58.5 cm vs. 60.7 cm, *p*=0.001) and head circumference (mean 39.6 cm vs. 40.5 cm, *p*=0.017) compared to the low ratio group. The perinatal outcome (**Table 4**) did not differ between the groups, except for ROP (*p*=0.022).

**Table 3:** Baseline patient characteristics.

Clinical characteristics	High sFlt-1/PlGF ratio (n=53)	Low sFlt-1/PlGF ratio (n=35)	*p^a^*
GA at birth, weeks	30.6 [3.6]	30.9 [4.3]	*0.973*
GA of ratio measurement, weeks	29.6 [3.8]	28 [4.7]	*0.100*
Difference GA ratio measurement and GA at birth, days	4 [6.5]	7 [11]	***0.048***
sFlt-1/PlGF Ratio	476.6 [903.3]	4.37 [16.4]	***< 0.001***
Child sex, male, n	24 (45.3%)	21 (60.0%)	*0.176*
Birth weight, g	1120 [687.5]	1550 [720]	***0.004***
SGA	20 (37.7%)	2 (5.7%)	***0.005***
Follow-Up characteristics			
Age at assessment, weeks	13 [1]	13 [1]	*0.098*
Weight at assessment, g	5450 [1485]	6200 [790]	***<0.001***
MOS-R, mean	22.3 [3]	22.0 [4]	*0.384*

Data are given as n (%) or median [IQR]. ^a^p values are calculated using Student’s t-test, Pearson’s chi-square test and Mann-Whitney U-test. SGA, Small for gestational age, defined as a birth weight ≤10 percentile.

Bold results indicate a p-value less than the significance level of 0.05.

**Table 4:** Perinatal outcome.

	High sFlt-1/PlGF ratio(n=53)	Low sFlt-1/PlGF ratio(n=35)	*p^a^*
**Morbidity**			
BPD	10 (18.9%)	4 (11.4%)	0.350
RDS	44 (83.0%)	31 (88.6%)	0.472
IVH > II	3 (5.7%)	4 (11.4%)	0.356
Hyperbilirubinemia	25 (47.2%)	14 (40.0%)	0.508
ROP	15 (28.3%)	3 (8.6%)	**0.022**
Sepsis	18 (34.0%)	7 (20.0%)	0.155
Hypoglycemia	16 (30.2%)	6 (17.1%)	0.167
**Breathing in the adaptation phase**			
spontaneous	6 (11.3%)	5 (14.3%)	0.681
Breathing support	41 (77.4%)	27 (77.1%)	0.981
Intubation	6 (11.3%)	3 (8.6%)	0.669
**Medication**			
Surfactant	27 (50.9%)	17 (48.6%)	0.827
Caffeine Citrate	36 (67.9%)	26 (74,3%)	0.522
Antibiotics	33 (62.2%)	18 (51.4%)	0.314

Data are given as n (%). ^a^p values are calculated using Pearson’s chi-square test and Mann-Whitney U-test. BPD, Bronchopulmonary dysplasia, RDS, Respiratory distress syndrome, IVH, Intraventricular hemorrhage, ROP, Retinopathy of prematurity.

Bold results indicate a p-value less than the significance level of 0.05.

**Figure 2:**
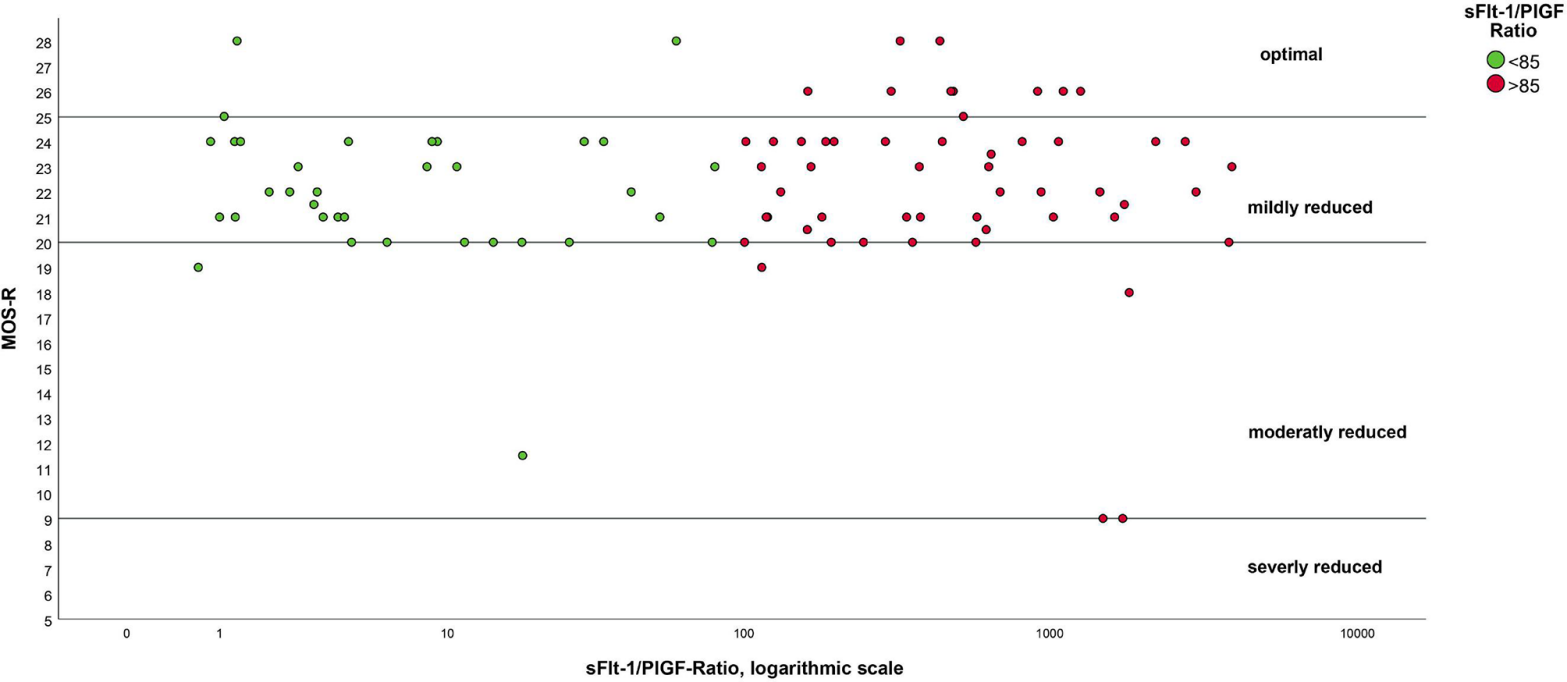
Results of MOS-R as a function of sFlt-1/PlGF ratio. Notes. The ratio has been logarithmized for better presentation.

A direct relationship between an increased sFlt-1/PlGF ratio and a worse motor outcome could not be demonstrated in linear regression analyses (β ≤0.001; *p=*0.282). The mean MOS-R did not differ between the two groups (22.3 vs. 22.0; *p=*0.384). All children with a maternal sFlt-1/PlGF ratio above 1400 did not achieve optimal results (MOS-R >25) in the MOS-R (**Figure 2**). The distribution in each subscale of the MOS-R was relatively balanced between the two groups (**Table 5**).

**Table 5:** MOS-R.

	High sFlt-1/PlGF ratio (n=53)	Low sFlt-1/PlGF ratio (n=35)	*p^a^*
**Fidgety Movements (FM)**			
Continual FM	41 (77.3%)	26 (74.3%)	*0.743*
Intermittent FM	10 (18.9%)	8 (22.8%)	*0.654*
Abnormal exaggerated	0	1 (2.9%)	*0.310*
Sporadic/absent FM	2 (3.8%)	0	*0.150*
**Movement patterns**			
Normal > abnormal	52 (98.1%)	35	*0.313*
Normal = abnormal	1 (1.9%)	0	*0.313*
Normal < abnormal	0	0	
**Movement repertoire**			
Age-adequate	16 (30.2%)	7 (20.0%)	*0.270*
reduced	23 (43.4%)	18 (51.4%)	*0.459*
Absent	14 (26.4%)	10 (28.6%)	*0.825*
**Postural patterns**			
Normal > abnormal	14 (26.4%)	10 (28.6%)	*0.825*
Normal = abnormal	18 (34.0%)	7 (20.0%)	*0.136*
Normal < abnormal	21 (39.6%)	18 (51.4%)	*0.274*
**Movement character**			
Smooth and fluent	9 (17.0%)	4 (11.4%)	*0.456*
Abnormal but not CS	43 (81.1%)	30 (85.7%)	*0.916*
Cramped-synchronized (CS)	1 (1.9%)	1 (2.9%)	*0.774*
**MOS-R**	22.3 [3]	22 [4]	0.384

Data are given as n (%) or mean [IQR]. MOS-R = Motor optimality score – revised. ^a^p values are calculated using Pearson’s chi-square test and Mann-Whitney U-test. sFlt-1= soluble Fms-like tyrosine kinase 1, PlGF = placental growth factor

**Figure 3:**
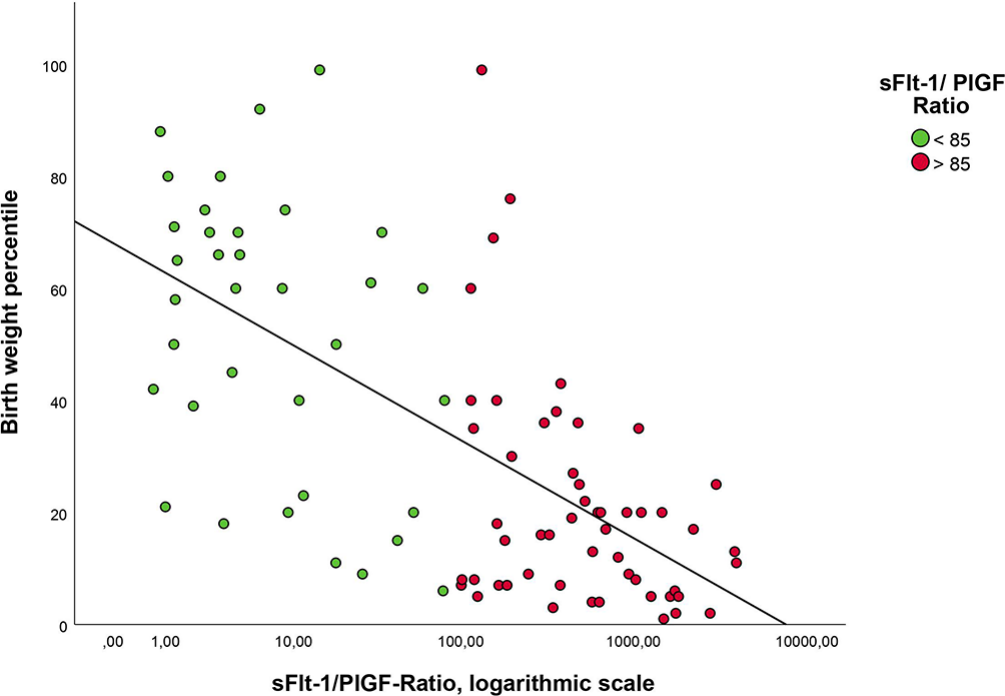
Correlation of birth weight percentile with sFlt-1/PlGF ratio. Notes. The ratio has been logarithmized for better presentation.

A significant difference was observed regarding the parameter of birth weight. The birth weight is negatively associated with the sFlt-1/PlGF ratio (β=-0.315; *p*<0.001) (**Figure 3**). Children born small for gestational age (SGA) (n=22), defined as birth weight below the 10th percentile, achieved significantly lower results in the MOS-R assessment (mean 20.7 vs 22.7; *p*=0.035). Of these children, 91% were in the group with an elevated sFlt-1/PlGF ratio. When considering the 38 FGR children in our cohort (which were not all born as SGA according to the definition mentioned above), they do not perform significantly worse in the MOS-R than the 50 non-FGR children (mean FGR: 21.8 vs. non-FGR: 22.5; *p*=0.209)

In the cohort with a high sFlt-1/PlGF ratio above 85 the mothers of the female offspring had lower average sFlt-1/PlGF ratios compared to the male offspring (median: 438 female offspring vs. 603 in male offspring, *p*=0.145). SGA was diagnosed in 46% of males in the high ratio cohort and 33% of females (*p*=0.269). BPD and ROP also affected the males slightly more often (*p*>0.05).

## Discussion

The aim of our study was to investigate the effects of increased maternal sFlt-1/PlGF ratio on early motor outcome of preterm infants by using the MOS-R. Overall, there were no significant differences in the MOS-R between the high sFlt-1/PlGF ratio (>85) group and the low ratio (<85) group. For the FM, specific to 3 months of age, there were also no differences between the two groups. A prediction of motor outcome based on the sFlt-1/PlGF-Ratio would be desirable but could not be demonstrated by our cohort.

However, this study demonstrated that children with a diagnosis of SGA scored worse on the MOS-R and thus represent a high-risk group for a worse motor outcome in our study. A total of 22 children were diagnosed with this condition. Of note, here 21 children of this cohort were diagnosed prenatally with FGR and 20 of them displayed an increased maternal sFlt-1/PlGF-Ratio.

This is consistent with the results of previous studies. A study by Sacchi et al. showed that FGR, very preterm birth (VPT) toddlers had significantly lower cognitive and motor scores in the 22-month Bayley Scales of Infant and Toddler Development III (Bayley-III) compared with appropriate for gestational age (AGA) VPT peers (16). Raz et al. examined fine and gross motor skills as well as performance IQ scores in a single center study of children born <34+6 GA, using the Peabody Developmental Motor Scales - Second Edition, at the age of 3 to 6 years. They could find poorer gross and fine motor skills for those with fetal growth restriction compared to those born with appropriate for GA birthweight. Moreover, they could demonstrate an association between intrauterine growth and motor skills as well as Performance IQ even in those who had adequate standardized birth weight (29). Deficits may continue into school age, as a study by Guellec et al., showed poorer outcomes of SGA children in terms of having a higher risk for minor cognitive difficulties, inattention-hyperactivity symptoms and school difficulties compared with AGA children (30).

SGA is mostly defined as a birth weight below the 10th percentile for the gestational age. This classification was originally developed by a 1995 World Health Organization (WHO) expert committee, and the definition is based on a birthweight-for-gestational-age measurement compared to a gender-specific reference population (31). The reduced birth weight is often caused by FGR, which in turn is associated with PE and placental insufficiency. The exact pathogenesis is still unclear but pro- and antiangiogenic factors seem to be key players. The overexpression of the angiogenic inhibitor sFlt-1 leads to an accumulation of sFlt-1 in maternal blood serum. sFlt-1 binds proangiogenic factors such as PlGF and VEGF before they have reached the membrane-bound receptor Flt-1 (fms-like tyrosin kinase 1), which would mediate proangiogenic signals to cells. In normal pregnancy, proangiogenic factors such as PlGF and VEGF lead to proliferation of endothelial cells, increased angiogenesis, and lower vascular resistance within the placenta (32, 33). The absence of proangiogenic signals leads to endothelial dysfunction and contributes to the development of PE (32, 34). It is known that newborns with maternal PE have increased sFlt-1 and decreased PlGF and VEGF values in the cord blood (35, 36). Whether and how these elevated sFlt-1 levels directly affect brain development is still the subject of current research.

Beside the development of PE, deficient pro-angiogenic factor expression is one aspect that may impair fetal vascularization, alter brain structure, and affect postnatal cognitive abilities of the offspring [28-30]

By using different transgenic mice models, it could be shown that loss of the pro-angiogenic factor PlGF, one of the two ligands of sFlt-1, could result in altered central nervous system vascularization, neuroanatomy, and behavior in mouse offspring (37, 38). Kay et al. found in their studies that the offspring of mice with PlGF deficiency had altered arteriovenous organization of the retinal vasculature (39). This was also reflected in our cohort, in which infants with a high maternal sFlt-1/PlGF ratio showed significantly more frequent retinopathy of prematurity. Delayed vascular organization in the retina could indicate a dysfunctional vasculature in other parts of the CNS and is a simple way to evaluate in infants. Some animal studies have investigated the impact of an anti-angiogenic environment on offspring cognition and behavior. In studies by Carver et al. using adenovirus-induced overexpression of sFlt-1, it was found that in male offspring the volume of the fimbria was smaller, and the volume of the neo-cortex was larger, whereas in female sFlt-1 offspring decreased volumes in the inferior colliculus, thalamus, and lateral globus pallidus was shown. Motor outcomes, testing vestibular function, balance, and coordination, were affected in both male and female offspring (40, 41). Collectively, all these mice studies assume that sFlt-1 overexpression resulted in a disturbed morphology in the fetal brain which may lead to short- and long-term neurocognitive disabilities.

While some data on the effects of an anti-angiogenic environment are already available for mice, the consequences for human infants are still mostly uninvestigated. In a study evaluating brain structural and vascular anatomy in 7- to 10-year-old offspring of preeclamptic pregnancies (with elevated maternal sFLT-1 serum levels) these children showed a significant enlargement of the cerebellum, brain stem, temporal lobe, and right and left amygdalae (42, 43). Additionally, a significant reduced vessel radii in the occipital and parietal lobes could also be revealed which may indicate a reduced blood flow to the fetal brain.

Chang et al. have already shown that infants with a high maternal sFlt-1/PlGF ratio above 85 had a lower birth weight (1142 ± 472 g vs. 2311 ± 236 g, p<0.001) and a higher risk for prematurity, BPD and respiratory distress syndrome (RDS) compared to the low ratio group (13). Also, Witwicki et al. demonstrated that children of women with a sFlt-1/PlGF ratio ≥33 were significantly smaller, had more respiratory and gastrointestinal morbidity, and longer hospitalization (44). A relationship between a high ratio and low birth weight was also shown in our study. However, we did not find a higher incidence of BPD, RDS or gastrointestinal diseases, which could be due to the fact that, in contrast to the studies above, we only included preterm infants in the study and that those complications are common in preterm infants in general. Like in our study, other previous studies have also been able to show an association between a high maternal sFlt-1/PlGF ratio and the frequency of SGA cases. In addition to its role as a predictor of SGA, the ratio may serve to differentiate between angiogenesis-dependent forms of SGA and other forms of SGA (45, 46).

In our study, in both groups, the most common score was in the “mildly reduced” range, which means a score between 20 and 24, most likely due to prematurity. This corresponds to the results of Salavati et al., 2021. In this study 180 very preterm and 180 healthy term infants participated. The median MOS-R scores of very preterm infants were significantly lower in comparison to those of term infants, with scores of 24 (25th-75th percentiles: 23-26) and 26 (25th-75th percentiles: 26-28), respectively (47).

Only from a sFlt-1/PlGF ratio above 1400 an optimal result (MOS-R 25-28 points) was not achieved, which applied to 11 children in our cohort (**Figure 3**). This could point to the fact that children with a very high maternal ratio might be less likely to have an optimal outcome.

Currently, there are no studies available to investigate the effect on the motor outcome of children born from mothers with a high sFlt-1/PlGF ratio. The existing studies focus on postnatal parameters such as APGAR or birth weight (13, 48). There is a lack of longitudinal studies as well as studies with larger patient collectives to evaluate other cofounders of outcome such as maternal sFlt-1/PLGF. Appropriate cofounders may be social environment and postnatal interventions in addition to biological factors (49–51).

In addition to the determination of angiogenic factors, ultrasonography was performed in the mothers. This included determination of the PI of the umbilical and uterine arteries and the CPR. The sFlt-1/PlGF ratio correlated significantly positively with PI of both uterine arteries, PI of the umbilical artery, and negatively with PI of the middle cerebral artery and CPR (36). In addition, all these parameters were worse in the high ratio mothers than in the control group. These results are consistent with previous studies on the relationship of sFlt-1 and PlGF to feto-maternal Doppler parameters (14). Increased uterine artery PI is associated with placental dysfunction and increased risk of FGR (52). During early stages of placental insufficiency, the CPR reflects fetal blood flow redistribution and is known to be an accurate predictor for fetal distress antepartum (53–55). A reduced CPR ratio has been associated by Monteith et al. with early childhood delayed neurodevelopment in the setting of FGR (56).

We used the cut-off of 85 for sFlt-1/PlGF ratio proposed by Verlohren et al., with a detection rate of PE of 88% and a specificity of 99.5% between 20 weeks and 34 weeks of gestation (8). However, our study revealed that the sFlt-1/PlGF-Ratio of 85 is not suitable to predict the motor outcome of preterm infants at 3 months of CA.

The present study has the following limitations: It must be kept in mind that 46% of the included infants in the control group with a low sFlt-1/PLGF ratio also exhibited postnatal morbidities during the clinical course, which can lead to reduced motor performance. These include BPD, sepsis, and hypoglycemia each of which may lead to impaired neurodevelopment and cognition (57–59). As discussed above, preterm birth is itself a risk for worse outcome in the MOS-R (47). Another limitation is partly incomplete existence of videos due to the following: Video analysis of motor function at 3 months of age was only gradually introduced in follow-up care from 2017. This has only been routinely done from 2018 onwards. In addition, some preterm infants born at Essen University Hospital were followed up in other follow-up centers. For some children, the videos were available, but maternal sFlt-1 and PlGF values were missing. This was because the values mentioned were often only collected in cases of suspected PE and not routinely or the prior care of the mothers took place in external practices. Since the MOS-R is an early diagnostic tool at 3 month of CA there is currently no long-term data for our cohort. However, the MOS-R at 3 months CA is known to predict overall adverse neurodevelopment, especially cerebral palsy, with high specificity and sensitivity (21).

Future follow-up of the cohort with evaluation of the Bayley Scales of Infant Development may provide further insight into long-term outcomes. In our cohort, children with a maternal sFlt-1/PLGF ratio above 1400 showed only non-optimal results in MOS-R. Increasing the size of the cohort would provide the opportunity to consider those infants with an extremely high maternal ratio separately.

## Conclusion

Early motor outcome of preterm infants, as measured by MOS-R, cannot be predicted by the level of the sFlt-1/PlGF ratio in the maternal serum in our study cohort. There were no differences between the two groups in the FM typical for the age of 3 months. However, this study showed that an increase of the sFlt-1/PlGF ratio is significantly correlated with lower birth weight and worse feto-maternal Doppler parameters. Especially low birth weight had a negative effect on the early motor outcome in the MOS-R.

## Data Availability Statements

The raw data supporting the conclusions of this article will be made available by the authors, without undue reservation.

## Ethics Statement

The studies involving human participants were reviewed and approved by Ethical committee, Universitiy of Duisburg-Essen, Essen, Germany (18-8388-BO, 12-5212-BO). Written informed consent to participate in this study was provided by the participants’ legal guardian/next of kin.

## Author Contributions

LM, BH and AK-D made the video analysis and infant data collection. BR and AI helped with measurements and maternal data collection. LM performed the data analysis and wrote the manuscript with support from BH, AK-D, AG, and IB. U-FM supervised the project. All authors contributed to the article and approved the submitted version.

## Funding

Funding was provided by German Research Foundation (DFG) [491780329] to AG and IB.

## Conflict of Interest

The authors declare that the research was conducted in the absence of any commercial or financial relationships that could be construed as a potential conflict of interest.

## Publisher’s Note

All claims expressed in this article are solely those of the authors and do not necessarily represent those of their affiliated organizations, or those of the publisher, the editors and the reviewers. Any product that may be evaluated in this article, or claim that may be made by its manufacturer, is not guaranteed or endorsed by the publisher.
